# Epidemiological characteristics of confirmed COVID-19 in Guizhou province, China

**DOI:** 10.1017/dmp.2020.134

**Published:** 2020-05-05

**Authors:** Xiahong Li, Xue Wang, Jun Liu, Guangtao Huang, Xiuquan Shi

**Affiliations:** Department of Epidemiology and Health Statistics, School of Public Health, Zunyi Medical University, Zunyi 563006, Guizhou, China; Affiliated Hospital of Zunyi Medical University, Zunyi 563099, Guizhou, China

**Keywords:** COVID-19, SARS-CoV-2, family-based transmission, migration index

## Abstract

**Objective:**

To explore the epidemiological characteristics of COVID-19 associated with SARS-Cov-2 in Guizhou province, and to compare the differences in epidemiology with other provinces.

**Methods:**

The data were extracted from National Health Commission of the People’s Republic of China, Health Commission of Guizhou province, and Health Commission of Hubei province from January 20 to February 12, 2020. Information included such as general demographic indicators, population data and clinical outcome.

**Results:**

A total of 135 cases were analyzed in the study. The average age was 39.46±18.95 years. The ratio of males to females was 0.985:1. Most of COVID-19 patients were 18-45 years old (52.27%). Close contact history was the most common (37.88%), followed by residence history in Hubei (34.85%). There was no difference between males and females in age (*P*=0.953) and exposure condition (*P*=0.186). Correlation analysis showed that there was a significant positive correlation between the migration index and the number of confirmed cases (r=0.816, *P*=0.007).

**Conclusion:**

Among the cases, most patients were young adults. Most epidemiological characteristics were no difference between males and females. Family-based transmission should not be ignored, as a close contact history was the top reason of exposure. Moreover, population movements also had significant impact on outbreaks.

## INTRODUCTION

Coronaviruses can cause zoonotic diseases, mainly severe respiratory infections in human^1^, which can be excreted through respiratory secretions, transmitted through oral fluids, sneezing, and contact, and transmitted through air droplets. Since the outbreak of Severe Acute Respiratory Syndrome coronavirus (SARS-Cov) that raged around the world in 2002-2003 followed by Middle East Respiratory Syndrome coronavirus (MERS-Cov) in 2012, coronavirus has received great attention from researchers and policy-makers around the world.^2^ Unexpectedly, a series of pneumonia cases of unknown cause emerged in Wuhan, the provincial capital city of Hubei province, China in December 2019. The etiology of the unknown pneumonia was “2019 novel coronavirus (2019-nCoV)” named by the World Health Organization (WHO) on January 12, 2020. It possibily was associated with wild animals from the seafood market in Wuhan where most of patients were exposed.^3-6^ However, human-to-human transmission has been responsible for most of the infections, including among health care providers.^4-6^ Unfortunately the 2019-nCoV has spread rapidly to different parts of China and at least 26 other countries.^7^ Moreover, the dramatic increase in the number of cases has caused widespread panic. On February 11, 2020, the International Committee on Taxonomy of Viruses (ICTV) announced that the official classification of the 2019-nCoV was renamed severe acute respiratory syndrome coronavirus 2 (SARS-CoV-2).^8^ Meanwhile the WHO announced that the official name of the disease caused by the virus was COVID-19.^9^

Guizhou province, located in a remote area of southwest China, has a population of 36 million, and the ratio of male to female is 1.07. The Gross Domestic Product (GDP) per capita of Guizhou province was $ 6,752 with ranking 29^th^ in China in 2019. The National Health Statistics Yearbook in 2019 showed that the urban population accounted for 47.53% of the total population in Guizhou^10^, which was lower than coastal areas in China. Some people have to work far away from hometown in terms of underdeveloped socio-economic conditions, some of whom go to work in Hubei province. Besides, Hubei province has many famous universities, which have attracted many students from Guizhou province to study. As those above reasons, people have more possibility of exposure to the SARS-CoV-2 during working and studying in Hubei. Additionally, the northern part of Guizhou province is almost adjacent to the southwest of Hubei province (Guizhou is not far from Hubei, the shortest distance is only about 100 kilometers).

**FIGURE 1:**
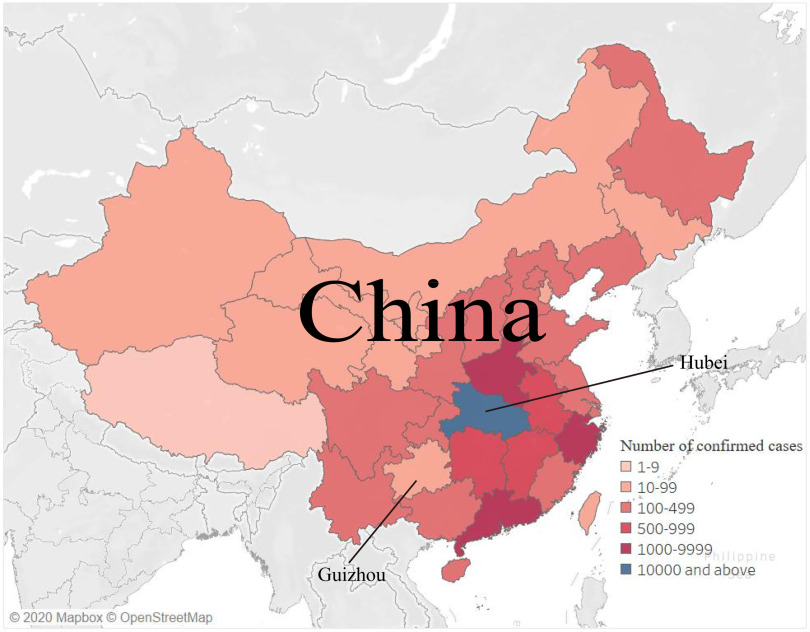
Epidemic distribution in China's provinces on February 8, 2020.

However, Since the epidemic situation outbreaks, the prevention and control situation in the provinces closer to Hubei is relatively difficulty, while there also appears a small exception in Guizhou province. The epidemic map shows that most of the provinces around Guizhou are more severe, and the accumulative numbers of diagnoses of Guizhou are even lower than all its neighbour provinces (Fig. 1). Therefore Guizhou province is valuable for researchers and the public to understand its uniqueness.

To the best of our knowledge, information regarding the epidemiological features of pneumonia caused by SARS-CoV-2 in Guizhou province is distinctive but scarce. In this study, we investigate the epidemiological characteristics of 135 patients confirmed to have SARS-CoV-2 infection, aiming to test whether SARS-CoV-2-infected patients may have different epidemiological characteristics than patients in other provinces, and provide an insight into the prevention and control of COVID-19 across China and even other places in the world.

## METHODS

### Data source

For this retrospective study, data were extracted from National Health Commission of the People’s Republic of China (http://www.nhc.gov.cn/), Health Commission of Guizhou province (http://www.gzhfpc.gov.cn/), and Health Commission of Hubei province (http://wjw.hubei.gov.cn/) for the time period from January 20 to February 12, 2020. We collected information, including general demographic indicators (such as age, gender, place of residence), population data (the accumulative cases, the number of accumulative rehabilitation, and the accumulative deathes), and clinical outcome (discharge, mortality). In addition, we also collected the migration index from Hubei province to all cities of Guizhou province from the migration map (also called as Baiduqianxi) of Baidu website (http://qianxi.baidu.com/) from January 6 to February 12, 2020. Because the incubation period for the COVID-19 is almost no more than 14 days, the data collection start time was January 6 (14 days before our collection start time of COVID-19). Finally, we needed to calculate the mean of daily migration index. In order to ensure the quality of the collected data, any duplicate or redundant information concerning the epidemic was cleaned.

### Inclusion criteria and exclusion criteria

All subjects with laboratory-confirmed SARS-CoV-2 infection who were reported by the local health authority were included in this study, and the data was available and complete without missing in some main fields for the time period from January 20 to February 12, 2020.

### Definition of study variables

Migration index is used to reflect the population movements from one city to another. In this study, the migration index was calculated as the population from Hubei province to all cities of Guizhou province divided by the total output population of Hubei province. This value could be extracted directly through the migration map without extra calculation.

### Statistical analysis

Microsoft Office Excel (version 2010) was used to build the database, and the data processing and analysis involved using SPSS software (Version 18.0, IBM Corp., Armonk, NY, USA). Categorical variables were expressed as number (%), and continuous variables expressed as mean and standard deviation (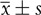). Chi-square tests were performed to assess the differences in categorical data. Correlation analysis was used to analyze correlation between the migration index and the number of confirmed cases. A two-side *P*-value of less than 0.05 was considered that the difference was statistically significant.

## RESULTS

### Epidemic distribution in China’s provinces

The epidemic map (Fig. 1) illustrated the epidemic distribution in each province in China on February 8, 2020. There were a total of 37198 admitted hospital patients were identified as laboratory-confirmed SARS-CoV-2 infection in China, of which the number of cases in Hubei were responsible for the highest proportion (72.85%), whereas these numbers were lower in Guizhou province than that of Hubei province and all other provinces around Guizhou.

**FIGURE 2:**
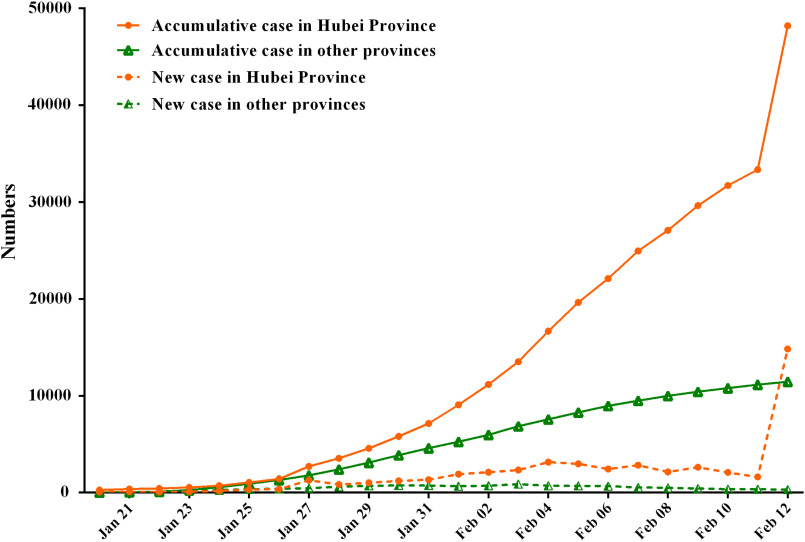
Time distribution of COVID-19 cases in Hubei province and other provinces.

**FIGURE 3:**
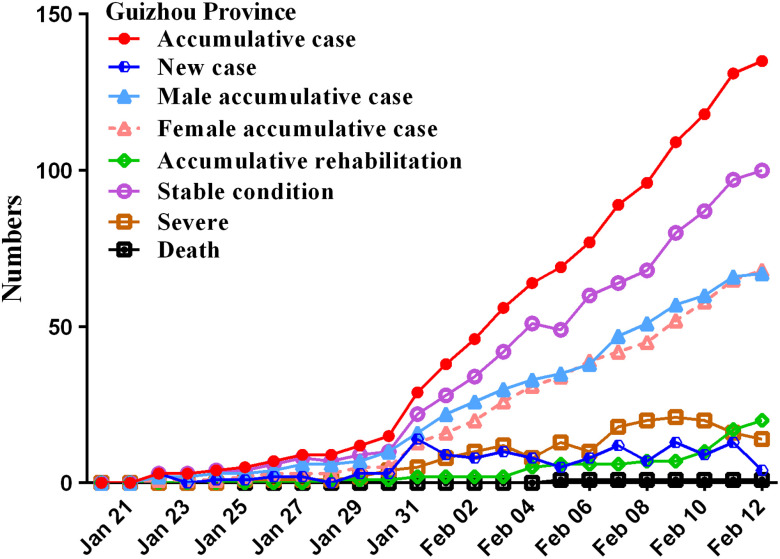
Time distribution of COVID-19 cases in Guizhou province.

In this study, by February 12, 2020, a total of 59,804 patients, of which 48,206 were in Hubei, 135 in Guizhou, and 11,463 in other provinces. From January 20 to February 12, the numbers of accumulative cases in China increased significantly, especially on February 12, the number of new cases rapidly reached 14,840 in Hubei province, whereas there was a trend of growth first and then decline in other provinces (Fig. 2), and these number increased and then remained relatively stable in Guizhou province (Fig. 3).

### General characteristics in Guizhou Province

**FIGURE 4:**
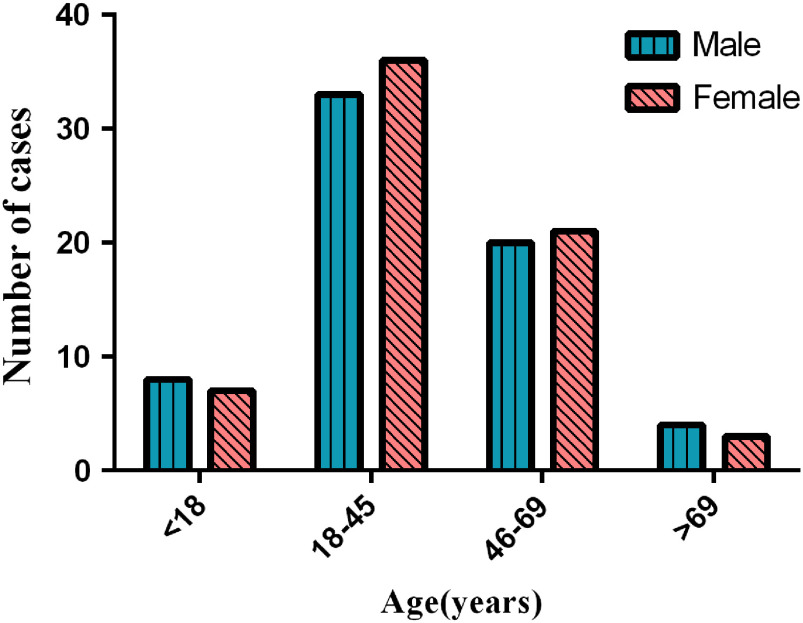
Age group distribution of COVID-19 cases in males and females.

In Guizhou province, a total of 135 cases were analyzed in the study (Three patients had incomplete information, so the sample size of some indices was only 132). The ratio of males to females was 0.985:1 (67 males/68 female) and showed no significant changes for the time period from January 20 to February 12. The average age was 39.46±18.95 years; most of the SARS-CoV-2 -infected patients were aged 18-45 years (52.27%), followed by 46-69 years (Table 1). In clinical outcome, 74.07% of the patients were stable condition, followed by 14.81% of the patients with rehabilitation (Table 1). There were 56.82% of patients going out of Guizhou before and during the epidemic, while it was not statistically significant differences between males and females. In exposure, close contact history was predomimant (37.88%), with residence history in Hubei province being the second most common (34.85%). According to Chi-square tests, there were no statistically significant differences in age and gender (Table 2 and Fig. 4).

**TABLE 1:** Epidemiological characteristics of COVID-19 in Guizhou[n (%)]

Characteristics	Cases [Number(%)]
**Age**	
<18	15(11.36)
18-45	69(52.27)
46-69	41(31.06)
>69	7(5.30)
**Gender**	
Male	65(49.24)
Female	67(50.76)
**Out of Guizhou**	
Yes	75(56.82)
No	57(43.18)
**Exposure**	
Residence history in Hubei	46(34.85)
Close contact history	50(37.88)
Other	36(27.27)
Clinical outcome*	
Rehabilitation	20(14.81)
Stable condition	100(74.07)
Severe	14(10.37)
Death	1(0.74)

Note: *: There were 3 patients having incomplete information, so the sample size of some indices was only 132 cases, while the clinical outcome (135 cases) was not missing.

**TABLE 2:** Epidemiological characteristics of males and females with COVID-19

Variable	Male(n_1_=65)	Female(n_2_=67)	Chi-square	*P*-value
Age				
<18	8(12.31)	7(10.45)	0.334	0.953
18-45	33(50.77)	36(53.73)		
46-69	20(30.77)	21(31.34)		
>69	4(6.15)	3(4.48)		
Out of Guizhou			1.163	0.281
Yes	40(61.54)	35(52.24)		
No	25(38.46)	32(47.76)		
Exposure			3.362	0.186
Residence history in Hubei	27(41.54)	19(28.36)		
Close contact history	20(30.77)	30(44.78)		
Other	18(27.69)	18(26.87)		

Moreover, the correlation was calculated between the migration index and the number of confirmed cases. The correlation coefficient was 0.816, *P*=0.007 (Table 3), which indicated that the migration index was significant positive correlation with the number of confirmed cases, and thus created a feasible univariate linear regression model: Ⓨ=7.859+69.855X+*e*. In the regression model, Ⓨ was the estimated value of confirmed COVID-19 number; X was the migration index (×100%), and “*e*” was the residual. The regression coefficient (B) was 69.855 (95% Confidence Interval [95% *CI*]: 25.660~114.051), which could explain when the migration index increased one percent on average, the confirmed COVID-19 cases should increase about 70 persons accordingly. The determination coefficient of the regression model was 0.666, which could explain 66.6% variation of the COVID-19 number in the linear model.

**TABLE 3:** Association between migration index and accummlative cases

City	Mean of daily migration index (%)	Accummlative cases[Number]	City	Mean of daily migration index (%)	Accummlative cases[Number]
Guiyang	0.321	33	Tongren	0.172	10
Zunyi	0.239	25	Qiannan	0.028	17
Anshun	0.004	4	Qianxi	0.000	4
Liupanshui	0.003	10	Qiandong	0.067	10
Bijie	0.086	22	Total of Guizhou*	0.920	135

* Correlation coefficient *r=*0.816; *P*-value=0.007

## DISCUSSION

The current SARS-CoV-2 outbreak in China is the third epidemic attributed to coronavirus in the 21st century, and incrediblely the number of confirmed SARS-CoV-2 infection has surpassed SARS and MERS.^6,7^ It has been declared as “Public health emergencies of international concern” by WHO on January 30, 2020. The higher number of cases possibly were relative to lack of etiological identification during the earliest period and human-to-human transmission, especially the individuals without symptom remain infectious ability.^5^ Moreover, as is well known, the speed of virus transmission may associate with the traffic condition. At the late January in 2020, it is peak of Spring Festival transportation (also named as “Chunyun” in China). Chunyun is a large-scale phenomenon of high transportation pressure that occurs in China around the Lunar New Year. This should accelerate the propagation of SARS-CoV-2. A research in Wuhan showed that clinical presentations of the patients with confirmed COVID-19 greatly resembled SARS-CoV and MERS, such as fever, dry cough, dyspnoea, whereas few patient devoloped obvious upper respiratory tract symptoms and intestinal infections.^4^

As of February 12, 2020, there were a total of 59,804 cases in China, of which Hubei prvince accounted for the highest proportion (80.61%). However, the 135 patients (0.23%) was included in this study, which were obviously lower in Guizhou than that of Hubei and even all other provinces around Guizhou. It might be associated with the following three reasons: Firstly, Guizhou, a predominantly mountain-rich region in China, has undeveloped traffic that leads to little population migration, therefore the incidence of SARS-CoV-2 infections was only 3.75/million. Secondly, Guizhou province expanded the detection scope from “only when suspected” to “preventive detection to any persons with a history of close contact” timely, and the possible source of infection has been detected and isolated without delay. Additionally, the epidemic prevention and control department took active response measures, for instance, on January 21, 2020, prevention and control systems were established at airports, stations, and docks, and ventilation and disinfection were performed on key public places. On January 24, a first-level response to a public health emergency was initiated. On January 26, the transportation hub strictly monitored the body temperature of pedestrian and suspended operations of public entertainment venues. Moreover, a total of 108 hospitals were designated for treatment, with 101,614 beds in general infection areas and 8,332 health care workers in Guizhou province.

Moreover, it was worth mentioning that the ratio of males to females was 0.985:1 in the 135 case of SARS-CoV-2 infection, which was inconsistent with previous studies from Wuhan^3^ and Zhejiang province^11^ that showed SARS-CoV-2 (former name 2019-nCoV) infection was prone to affect males. The possible reason was that the confirmed COVID-19 in Guizhou province appeared an obvious family aggregation, so the prevalence of SARS-CoV-2 infection was almost balanced in gender in Guizhou than other provinces. Besides, the gender difference might depend on whether suspected cases were receptive to care access, and whether they sought for medical support from different areas. Our study showed that most of the infected patients were young adults(52.27%), which was different from the result of Chen et al.^3^ The possible explanation was that young adults need to go out to work and study, while older and children are mostly at home. In this study, by the end of February 12, only one death toll was reported; the death was a 34-year-old male with a Wuhan travel history. He had hypertension underlying disease. The result was consistent with the recent report that the comorbidity as a consequence of poor immune fuction might be a risk factor for undesirable outcome.^12^

Some studies suggested that rapid person-to-person transmission between close contacts was an important feature of SARS-CoV-2 pneumonia.^11-13^ Additionally, some researches about impact of cases exported from Hubei province on accumulative cases in different provinces of China showed that the accumulative cases of provinces outside Hubei province were positively correlated with the migration index derived from Hubei province.^14,15^ Our study also showed that there was a positive correlation between the migration index and the number of confirmed cases, and the determination coefficient of the regression model could explain 66.6% variation of the number of COVID-19, which suggested a significant influence of population movements on outbreak of COVID-19. Unfortunately, our model mainly targeted on the first generation cases who were migrated from Hubei to Guizhou. As in the later stage of COVID-19 epidemic, most patients were clustered and had a history of close contact, and spread the SARS-CoV-2 to family members and relatives in asymptomatic conditions, so COVID-19 predictive number by our linear regression model might be biased.

### Limitations of this study

This study should be carefully interpreted because of the following limitations. Firstly, the information about clinical data of 135 patients with confirmed COVID-19 in Guizhou Province was insufficient, therefore, we could not analyze the clinical characteristics of patients in more details. Secondly, as this study was retrospective there were both time lag and observation biases in our research. Finally, our study was limited to singlecentre study in Guizhou province, therefore the sample size might not be enough. It would be better to involve as many as possibie in other provinces of China, and even in other counties to build a large sample and multicenter study about COVID-19.

## CONCLUSIONS

In conclusion, most of the infected patients was young adults, and there were no statistically significant differences between males and females in the number of confirmed COVID-19 cases. In addition, most patients had a history of close contact, especially family-based transmission, and population movements also played a crucial factor on outbreaks.

## Supplementary material

For supplementary material accompanying this paper visit https://doi.org/10.1017/dmp.2020.134. click here to view supplementary material
